# Current management strategies for visceral artery aneurysms: an overview

**DOI:** 10.1007/s00595-019-01898-3

**Published:** 2019-10-16

**Authors:** Hideaki Obara, Matsubara Kentaro, Masanori Inoue, Yuko Kitagawa

**Affiliations:** 1 Department of Surgery, Keio University School of Medicine, 35 Shinanomachi, Shinjuku-ku, Tokyo, 160-8582 Japan; 2 Department of Diagnostic Radiology, Keio University School of Medicine, Tokyo, Japan

**Keywords:** Visceral artery aneurysm, Pseudoaneurysm, Aneurysm rupture, Endovascular therapy

## Abstract

Visceral artery aneurysms (VAAs) are rare and affect the celiac artery, superior mesenteric artery, and inferior mesenteric artery, and their branches. The natural history of VAAs is not well understood as they are often asymptomatic and found incidentally; however, they carry a risk of rupture that can result in death from hemorrhage in the peritoneal cavity, retroperitoneal space, or gastrointestinal tract. Recent advances in imaging technology and its availability allow us to diagnose all types of VAA. VAAs can be treated by open surgery, laparoscopic surgery, endovascular therapy, or a hybrid approach. However, there are still no specific indications for the treatment of VAAs, and the best strategy depends on the anatomical location of the aneurysm as well as the clinical presentation of the patient. This article reviews the literature on the etiology, clinical features, diagnosis, and anatomic characteristics of each type of VAA and discusses the current options for their treatment and management.

## Introduction

Visceral artery aneurysms (VAAs) are rare and usually asymptomatic, but can prove fatal if they rupture. In 1770, Beaussier reported a case of VAA in a 60-year-old woman from France, whose autopsy led to the discovery of a splenic aneurysm [1]. In the 1900s, there were reports of successful surgical treatment of traumatic hepatic aneurysms [2]. In 1953, Debakey and Cooley [3] reported a case of an infected superior mesenteric artery aneurysm (SMAA), since when VAA has appeared in the literature. Recent advances in computed tomography (CT) and other diagnostic imaging modalities, as well as remarkable developments in endovascular therapy (EVT) have improved the diagnosis and treatment of VAA, resulting in the release of the 2017 European Society for Vascular Surgery (ESVS) guidelines [4].

We review the literature on the etiology, clinical features, diagnosis, and anatomic characteristics of each type of VAA and discuss the current options for its treatment and management.

## Incidence and etiology

VAA was defined in this study as an aneurysm in the celiac artery, superior mesenteric artery (SMA), inferior mesenteric artery, and/or their branches. Aortic and renal artery aneurysms, which account for approximately 95% of all aneurysms in the abdominal cavity, were not included. Approximately, one-third of patients with a VAA have comorbid aneurysms in other visceral arteries, the aorta, the iliac artery, the renal arteries, and arteries in the lower extremities or intracranial arteries [4]. In 2018, Erben et al. [5] reported that concomitant aneurysms elsewhere in the vascular tree were identified in 46% of patients with VAAs, including the renal artery (14%) and other visceral arteries (12%). Based on autopsy results, the incidence of VAA is between 0.01 and 2% [6–9]; however, the widespread use of CT has increased the incidental diagnosis of asymptomatic VAAs.

Studies conducted in the 1970s by Stanley et al. [10] and Deterling et al. [11] reported that 60% of VAAs were splenic artery aneurysms (SAAs), 20% were hepatic artery aneurysms (HAAs), 5–8% were SMAAs, 4% were celiac artery aneurysms (CAAs), 2–4% were gastric or gastroepiploic artery aneurysms (GEAAs), 2–3% were jejunal, ileal, and colic artery aneurysms, and 2% were pancreaticoduodenal artery aneurysms (PDAAs). In more recent studies, 30–36% were SAAs, 2–46% were CAAs, 4–30% were HAAs, 2–10% were PDAAs, 3–9% were SMAAs, 1–15% were gastroduodenal artery aneurysms (GDAAs), 2% were GEAAs, 1% were inferior mesenteric artery aneurysms (IMAAs), and 3–6% were jejunal, ileal, and colic artery aneurysms [5, 7, 12]. In previous case reports and reviews, the incidence of each aneurysm site in the visceral artery aneurysms depended on whether renal artery aneurysms were included in the definition of VAA, whether cases that were followed up and treated were included, and whether iatrogenic cases were included. Despite these differences, SAA is established as the most common type of VAA.

Histopathologically, VAAs are broadly divided into true aneurysms and pseudoaneurysms. True aneurysms are those that expand locally while maintaining components of the arterial wall, and their main cause is atherosclerosis. Other possible causes of true aneurysms include connective tissue diseases and fibromuscular dysplasia [13–15]. Conversely, pseudoaneurysms are attributed to a lack of arterial wall structure usually following trauma, infection, vasculitis, inflammation, or iatrogenic causes. It has been recognized recently that the trend of performing invasive procedures for hepatobiliary diseases has increased the frequency of iatrogenic pseudoaneurysms [16]. Moreover, the number of studies on segmental arterial mediolysis (SAM) has been increasing. SAM, which is a rare, nonatherosclerotic, nonvasculitic arteriopathy, characterized by the development of a dissecting hematoma, aneurysm, occlusion, or hemorrhage after lysis of the arterial media, usually involves more than one visceral artery [17–19].

VAA is also found in conjunction with rare diseases such as von Recklinghausen disease, Ehlers–Danlos syndrome, periarteritis nodosa, and Behçet disease [14]. Morphologically, it is categorized as either a fusiform or saccular aneurysm, and pathologically, it is categorized as ruptured or unruptured.

## Diagnosis

VAAs are usually silent, but potentially fatal because of the risk of rupture. Even after rupture, the symptoms such as abdominal and back pain and anemia in some patients are tolerable, which can make VAAs difficult to diagnose. Therefore, VAA should be considered in the differential diagnosis when a patient presents with sudden abdominal pain accompanied by anemia. In addition to diagnosis following rupture, other cases are found incidentally during detailed investigations for unrelated diseases or screenings using ultrasound (US) and CT. Contrast-enhanced CT angiography, magnetic resonance angiography, and abdominal US help identify the morphology and features of the aneurysm, and thereby facilitate a definitive diagnosis. In addition to conventional axial CT images, images reconstructed using volume rendering and multiplanar reconstruction based on data obtained from multidetector row CT provide 3D data that are useful for planning a treatment strategy. Although angiography is more invasive and requires more time than the non-invasive imaging modalities, it can provide a detailed rendering of the blood vessels and allows the physician to focus immediately on treatment when the image indicates imminent rupture, making it invaluable.

### Treatment options

Rupture and bleeding are the most critical symptoms of VAAs, but because of their rarity, the natural course of VAAs remains unclear. The location, characteristics, size, and patient background characteristics affect the risk of rupture [14, 20]. In early reports, 25% of cases were of ruptured aneurysms [21], and the mortality rate ranged from 25 to 70% [22, 23]. Recent studies suggest that most asymptomatic true aneurysms increase to 20–25 mm in diameter slowly, indicating that they have a low risk of rupture and a good prognosis, which provides the opportunity to observe the disease course. Therefore, asymptomatic true aneurysms larger than 20–25 mm in diameter will require treatment [4, 5, 12, 24]. Conversely, aneurysms in the pancreaticoduodenal arcade, gastroduodenal aneurysms, and HAAs have a higher risk of rupture regardless of their morphology, making proactive treatment necessary, irrespective of the size of the aneurysm [4, 5, 12]. There is also a risk of rupture when the aneurysm is associated with inflammation, infection, connective tissue disease, vasculitis, or congenital disorders such as Marfan syndrome, Ehlers–Danlos type IV, or von Recklinghausen disease [25]. Treatment of pseudoaneurysms is mandatory because of the high risk of rupture [4, 7, 20, 25, 26]. Rupture of an SAA during pregnancy is fatal to both the mother and the fetus in 70% and 90–95% of cases, respectively [24, 27]. Therefore, VAAs diagnosed in women who may be pregnant need intervention [4, 24]. Portal hypertension is also a risk factor for rupture, so treatment is indicated regardless of aneurysm size in patients waiting for liver and other abdominal organ transplants [24, 25, 28–30]. There is no firm evidence that aneurysm calcification is protective against rupture [31].Once it has been established that treatment is indicated, detailed investigation of image findings is required to determine the appropriate treatment strategy.

**Fig. 1:**
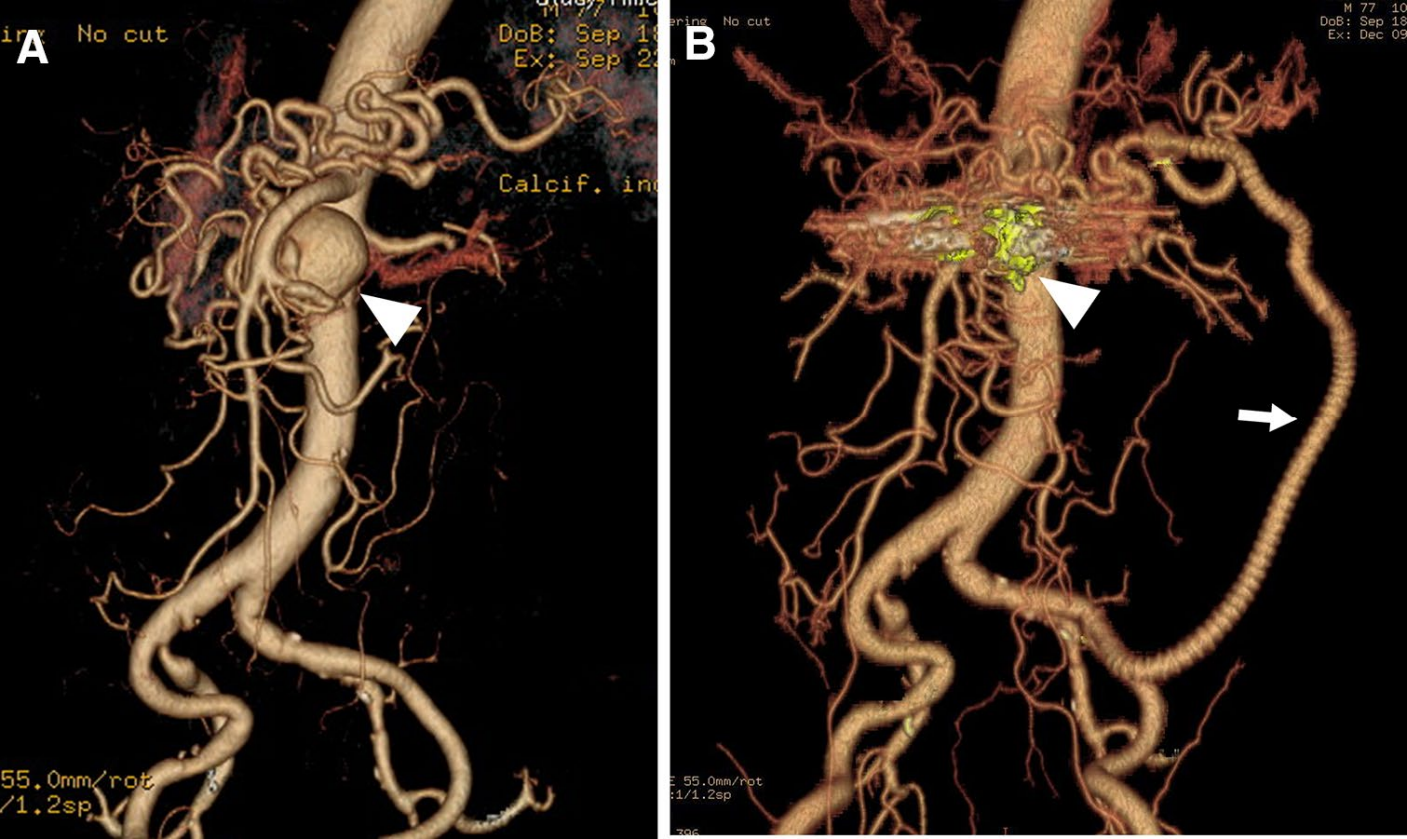
Inferior pancreaticoduodenal artery aneurysm in 77-year-old man. **a** Three-dimensional volume rendered computed tomography (CT) angiography shows a saccular aneurysm (35 mm) of the inferior pancreaticoduodenal artery (IPDA), adjacent to the origin of the IPDA with celiac axis occlusion. The main blood flow to the liver and spleen was supplied via this IPDA aneurysm. **b** Postoperative three-dimensional volume rendered CT angiography shows the successful coil embolization (arrowhead) and the patent prosthetic graft (arrow) from the iliac artery to the dorsal pancreatic artery to preserve organ blood flow

Various therapeutic strategies are available for VAAs, including open surgery (aneurysmectomy, ligation, and bypass with a venous graft or prosthetic vascular graft), laparoscopic surgery (mainly ligation), EVT (embolization and endografting), or a combination of treatments (hybrid approach) (Fig. 1), depending on the anatomical characteristics such as size, site, and features, and the etiology of the aneurysm, as well as the patient’s presentation, comorbidities, and risk factors. The selection of a specific VAA treatment also depends on the capability of each facility and individual preference. Thus far, no randomized control studies or prospective studies have compared the efficacy of different treatment strategies for patients with VAAs. Consequently, there is limited evidence about the effectiveness of these treatment strategies for VAAs. There is no consensus about the optimal management for VAAs, and current management strategies suggested in the literature are based on observational studies and case series [4, 14, 25, 30]. Despite the limited evidence from previous retrospective studies, it is known that peri-procedural complications are more likely after open repair. Recently, Barrionuevo et al. [23] conducted a systematic review and meta-analysis and suggested that EVT is associated with shorter hospital stay and lower rates of cardiovascular complications. These results may justify the endovascular approach as a first-line treatment. Furthermore, following rupture, the benefit of EVT seems to be greater, as open repair is more complex and has higher morbidity [32].

### Endovascular therapy (EVT)

**Fig. 2:**
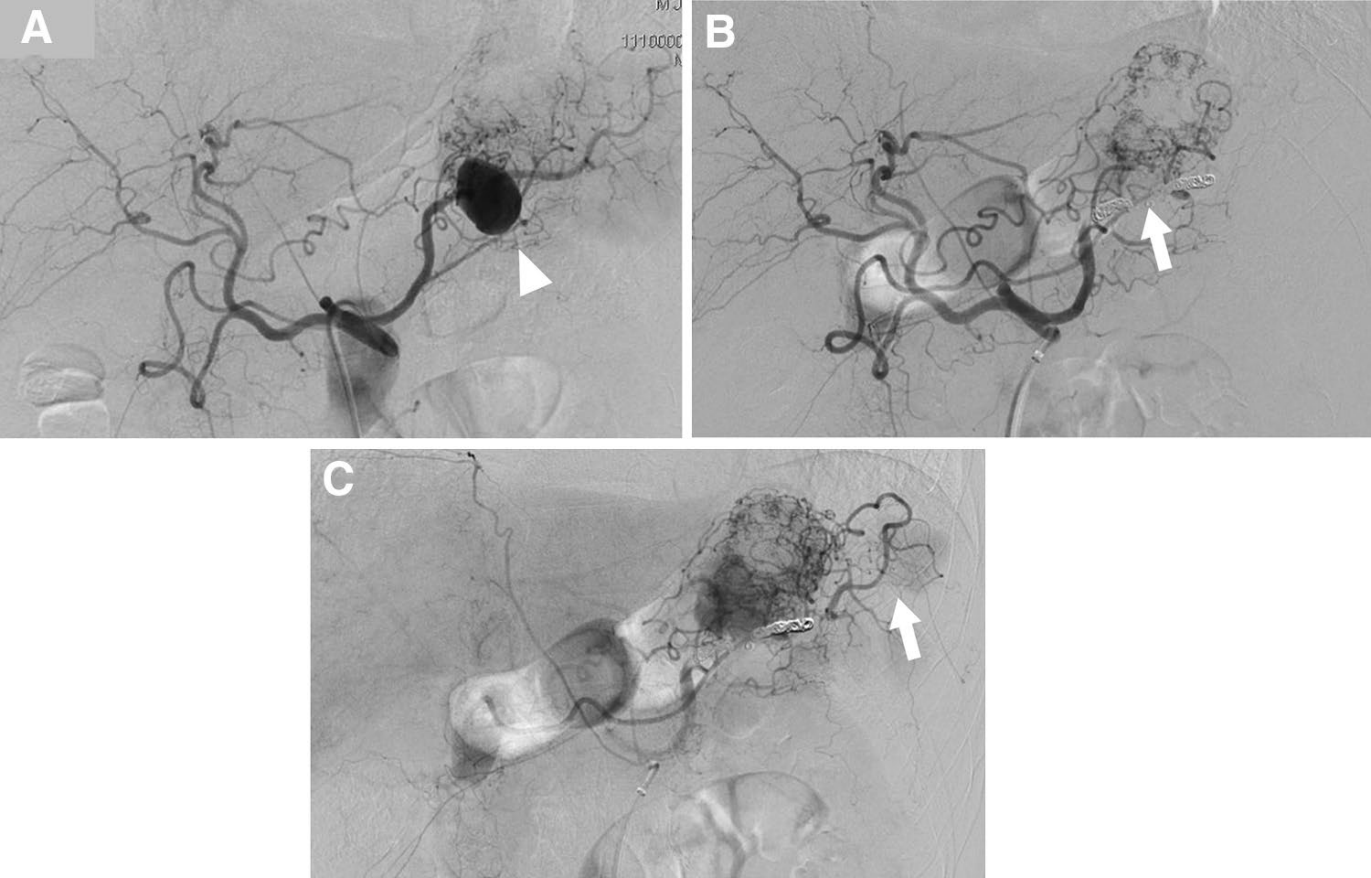
Endovascular coil embolization of the splenic artery aneurysm using an isolation technique. **a** Selective angiography of the celiac artery shows a large splenic artery aneurysm (arrowhead). **b** Celiac arteriography following microcoil embolization of the outflow and inflow arteries shows complete eradication of the aneurysm (arrow). **c** The late phase of the celiac artery arteriography shows the collateral blood supply to the spleen (arrow)

**Fig. 3:**
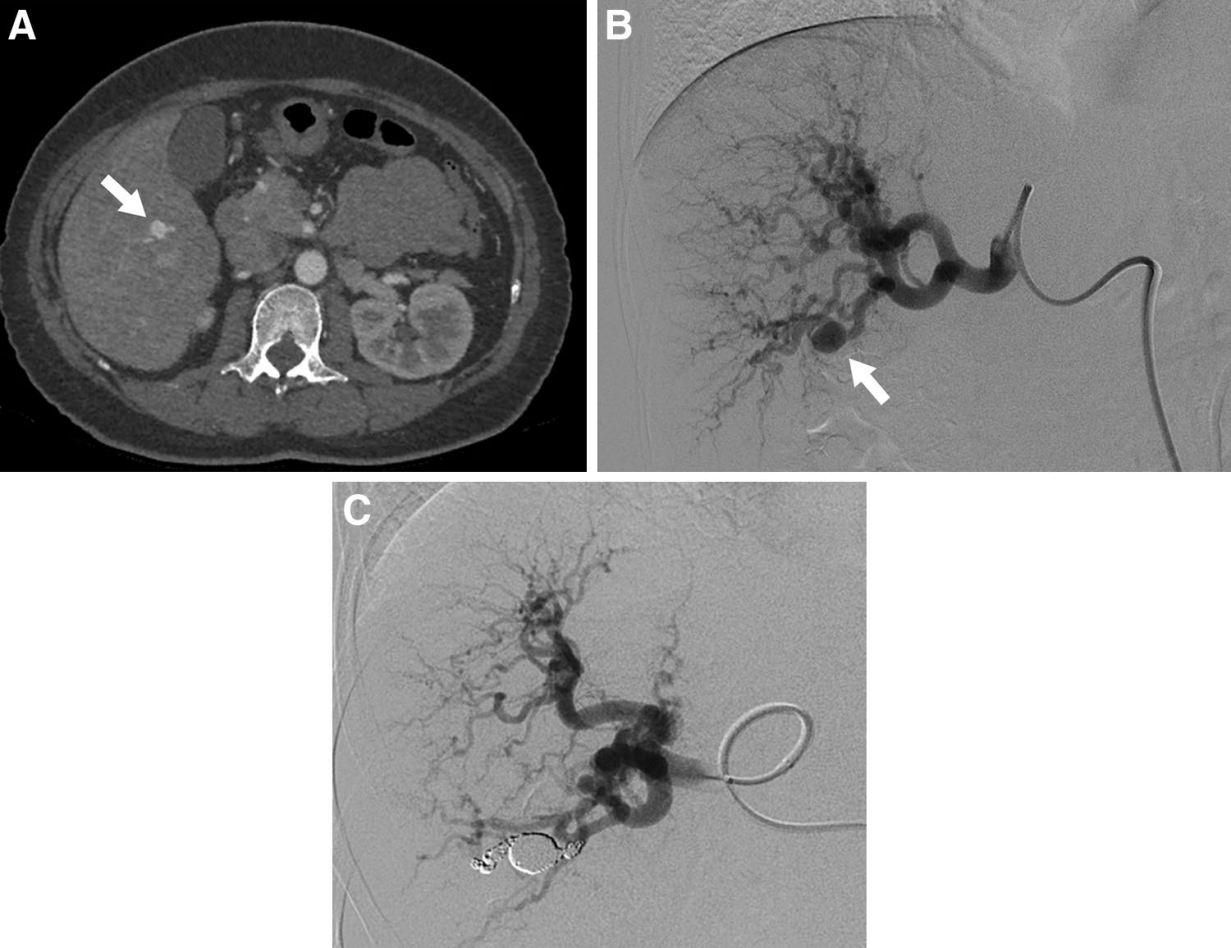
Hepatic artery aneurysm in 53-year-old woman with hereditary hemorrhagic telangiectasia. **a** The arterial phase of a contrast-enhanced CT scan reveals a hepatic artery aneurysm (arrow) located in the posterior segment of the liver. **b** Digital subtraction angiography of the hepatic artery demonstrates a saccular aneurysm (arrow) in the posterior branch of hepatic artery. **c** Postprocedural hepatic digital subtraction angiography shows complete obstruction of the aneurysm as well as the inflow and outflow arteries

**Fig. 4:**
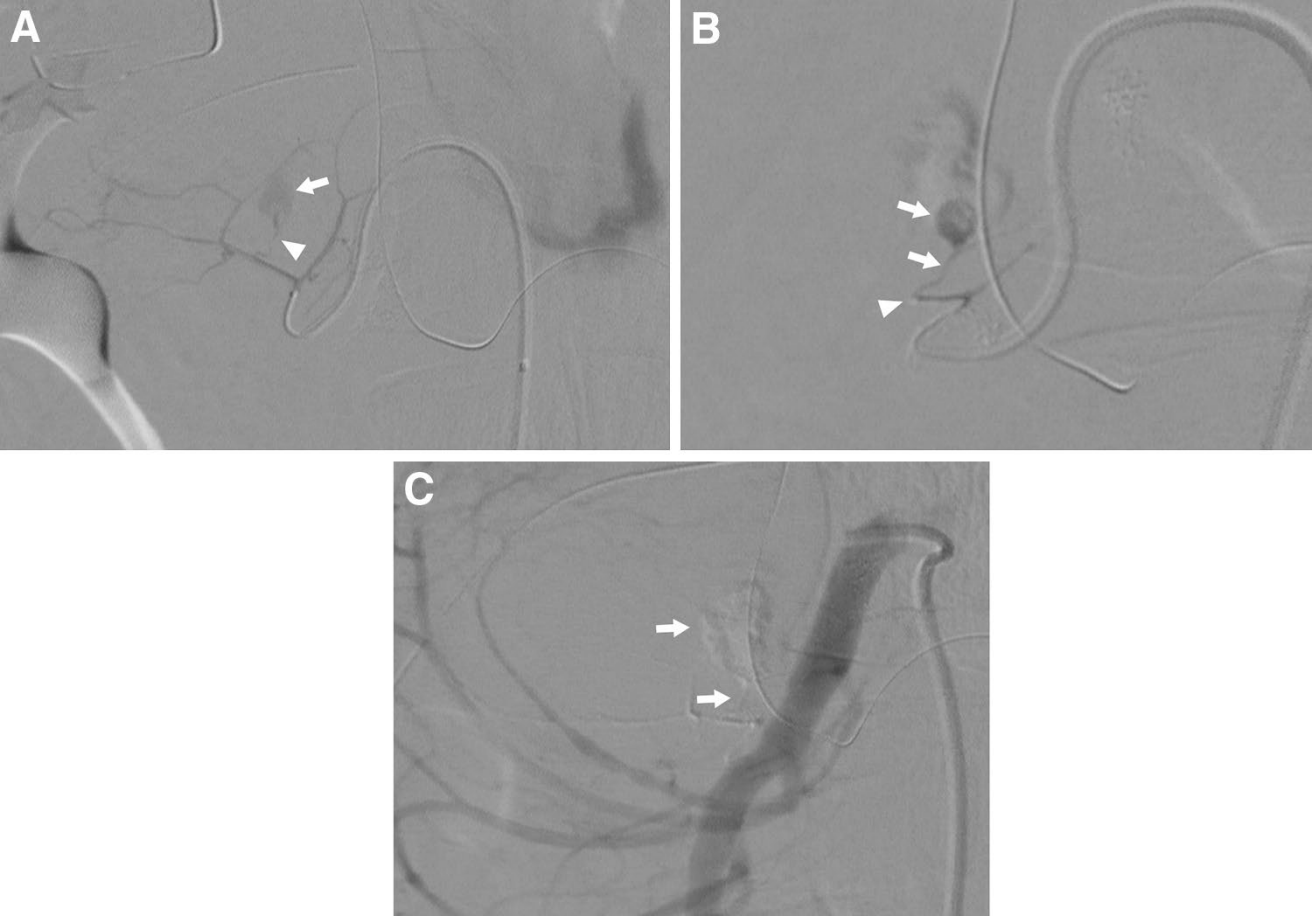
Pseudoaneurysms in 56-year-old man post-pancreaticoduodenectomy were treated using a liquid embolic agent. **a** Digital subtraction angiography from a branch of the jejunal artery reveals a pseudoaneurysm (arrow) in a tiny branch (arrowhead). **b** The pseudoaneurysm and the jejunal branch are filled with the liquid embolic agent (NBCA: *N*-butyl-2-cyanoacrylate) (arrow) from a microcatheter. An arrowhead shows the tip of the microcatheter. **c** Post-procedural superior mesenteric digital subtraction angiography shows complete embolization of the pseudoaneurysm and the feeding artery (arrows) with NBCA

One of the major benefits of EVT is that it is minimally invasive. It can be performed using local anesthesia and is followed by early postoperative recovery and consequently, a shorter hospital stay. Thus, EVT is effective for high-risk patients with multiple comorbidities and those with a history of abdominal surgery, for whom intraperitoneal adhesion is a concern. The primary limitations of EVT are the lack of availability of facilities with adequate resources for emergency treatment, the risk of access-related injury and end-organ embolization, contrast toxicity, and the need for prolonged imaging surveillance. EVT can be achieved by various techniques using a combination of different modalities, including deployment of coils and vascular plugs, injection of liquid embolic agents, placement of covered stents or flow-diverting stents, and percutaneous injection of thrombin [33]. Selection of the technique is based on the necessity to preserve the parent artery, the anatomic shape and type of aneurysm (narrow neck or fusiform/wide neck), and the tortuosity of the parent artery [30, 33]. Embolization techniques include embolization of a distal artery, followed by coil packing of the arterial aneurysm, and finally embolization of a proximal artery. If branches can be seen on the wall of the aneurysm, these can be embolized selectively. For pseudoaneurysms that have caused the blood vessel to rupture, an isolation technique with embolization of the arteries distal and proximal to the aneurysm is effective [33]. Successful isolation of the aneurysm is achieved only by complete embolization of all inflow and outflow arteries (Fig. 2). In many cases, microcoils are useful in the embolization of VAAs, but if the diameter of the target artery is larger than 3 mm, 0.035-inch metallic coils and vascular plugs are effective [33]. For aneurysms that develop in a terminal branch, the afferent artery can be embolized directly by coils (Fig. 3) or liquid embolic agents [32]. Endovascular coil embolization is a relatively easier procedure that has achieved good results [20, 25, 26, 33, 34]. Embolization using liquid embolic agents can also be effective, especially for complex VAAs, including pseudoaneurysms, but it is technically challenging due to the risk of distal embolization (Fig. 4) [20, 35, 36].

**Fig. 5:**
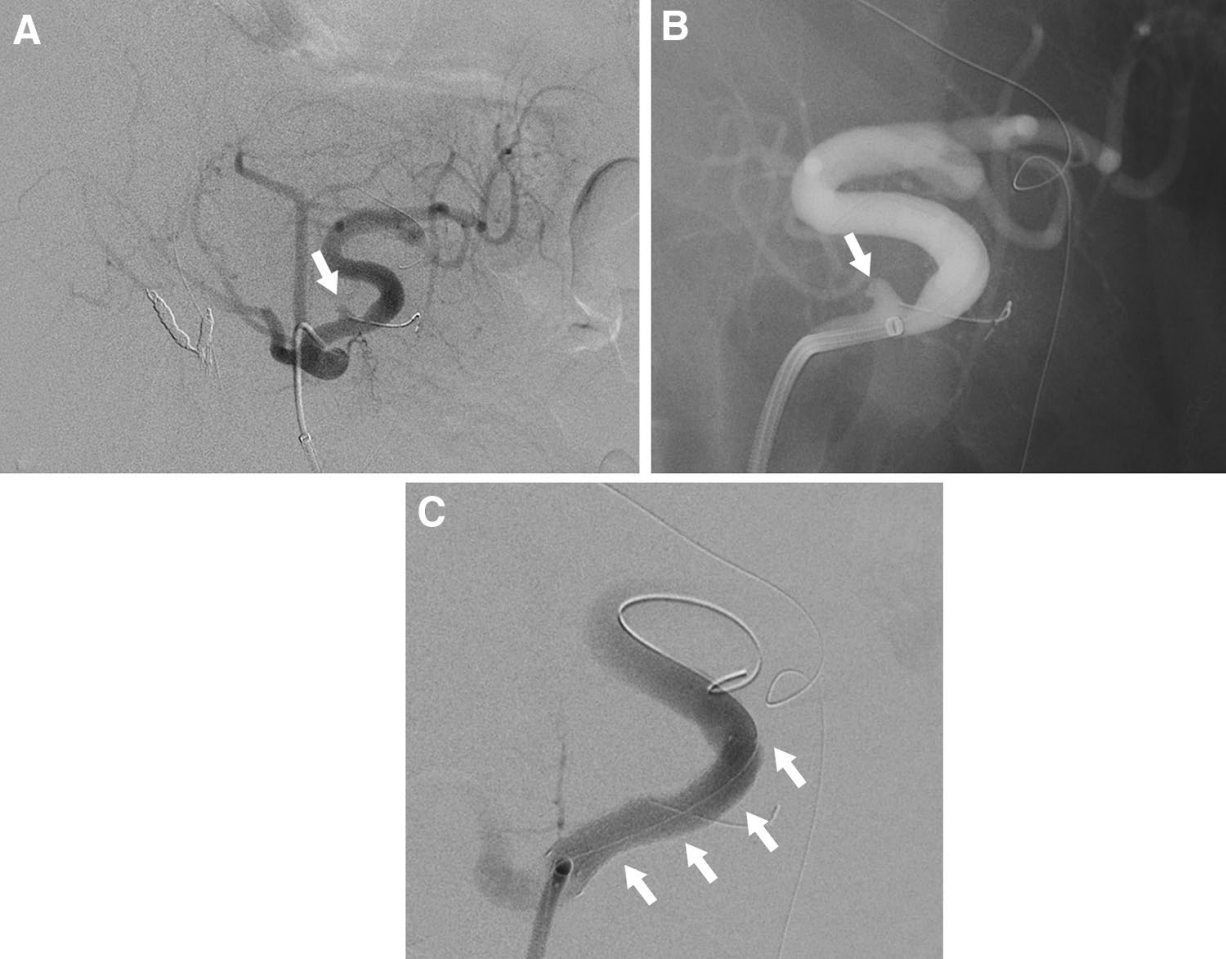
Splenic artery pseudoaneurysm in a 69-year-old man post-pancreaticoduodenectomy. **a** Digital subtraction angiography of the celiac trunk shows a pseudoaneurysm (arrow) in the proximal part of the splenic artery. **b** Selective splenic artery angiography shows a pseudoaneurysm (arrow) in the proximal part of the splenic artery. **c** Postprocedural subtraction angiography shows complete eradication of the bleeding pseudoaneurysm, with accurate stent-graft placement (arrows) and good patency of the splenic artery

In selective cases, a covered self-expanding stent can be placed to preserve the perfusion of blood to the distal target organ [37–39]. According to a report on EVT of 44 self-expandable stent graft procedures in 40 patients performed for 16 true VAAs and 24 pseudoaneurysms, there were 19 elective procedures and 25 emergency procedures performed to manage ruptured aneurysms [39]. The overall technical and clinical success rates were 96% and 84%, respectively, whereas the technical and clinical success rates for the 19 elective treatments were both 100%. Another study showed excellent long-term outcomes and a high patency rate [38]. Covered stent placement may also be effective for iatrogenic pseudoaneurysms in the gastroduodenal artery (GDA), splenic artery, common hepatic artery, or proper hepatic artery after pancreaticoduodenectomy, since simple coil embolization of these arteries carries a high risk of liver failure caused by blood flow disruption (Fig. 5) [40]. However, this technique, even if conceptually feasible, is often limited by the arterial anatomy and location of the aneurysm, since relatively large and rigid delivery devices have to be navigated to the target artery. As more flexible stent graft and smaller delivery systems are being developed with advances in endovascular technology, this technique may be applicable for more cases [32].

### Open repair

Open surgical repair of VAAs is a safe and durable standard treatment option. The primary benefit of open surgery is that it allows clear visual confirmation of the condition of the end organ. Therefore, the need for revascularization can be confirmed and the results checked during the surgery. Nevertheless, with advances in and expanded indications for EVT, open surgery tends to be selected only when EVT is too difficult to perform. Proximal and distal ligation of the VAA without arterial reconstruction has been used widely in emergency conditions, such after frank rupture, and in elective cases if collateral circulation is adequate [14, 41]. Since the spleen has abundant collateral circulation, simple ligation of the main trunk of the splenic artery does not cause major complications. Aneurysmectomy with end-to-end anastomosis can be performed safely. If the SPAA is located in one-third of the peripheral region of the artery or in the vessel that branches off the splenic hilum, then vascular reconstruction is difficult, in which case, splenectomy is usually performed. The celiac artery and common hepatic artery can also be ligated if there is sufficient collateral circulation from the gastroduodenal and pancreaticoduodenal arteries. The use of a vein graft for arterial reconstruction is recommended in the presence of infection or intestinal ischemia. Nevertheless, simple arterial ligation is a feasible procedure to consider for free rupture [22].

### Specific visceral artery aneurysms

#### Splenic artery aneurysms

**Fig. 6:**
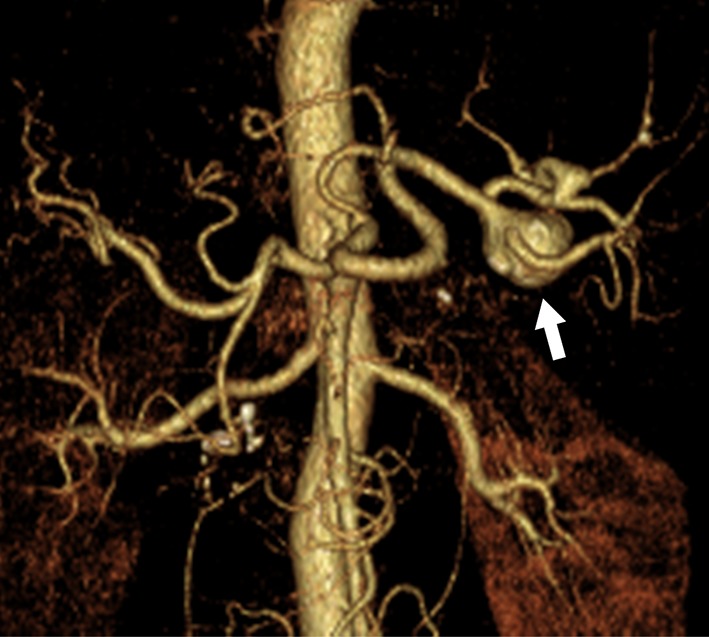
Splenic artery aneurysm in a liver transplant recipient. Three-dimensional volume rendered CT angiography shows a saccular aneurysm in the distal splenic artery aneurysm (25 mm) in a liver transplant recipient (arrow). Splenectomy was performed at the time of liver transplantation

SAAs account for nearly 60% of reported VAAs. Most SAAs have saccular morphology and are located in the middle to distal splenic artery [25]. In contrast to other arterial aneurysms, SAAs have a 4:1 female-to-male predominance and are often found in multiparous women [25]. It is posited that hormonal changes during pregnancy may be associated with aneurysm formation and rupture, but the precise mechanisms are not fully understood [25, 42]. Other associated risk factors for a true aneurysm include portal hypertension, fibromuscular dysplasia, SAM, and atherosclerosis [15, 25, 29, 43]. Crons et al. [28] reported that SAAs were identified in 20.5% of liver transplant recipients (Fig. 6). Moreover, pseudoaneurysms can develop secondary to pancreatitis or trauma [44]. Hemosuccus pancreaticus, generally the result of rupture of a SPAA into the pancreatic duct, is a rare cause of intermittent upper gastrointestinal hemorrhage [18]. Most SAAs are asymptomatic and found incidentally on imaging scans performed for unrelated symptoms or during screening for other diseases. SAAs not associated with pregnancy have a relatively low rupture rate (< 2%) with an associated mortality rate of 36% [45]. The risk of rupture is high when the SAA is associated with pregnancy (maternal mortality rate, 70%; fetal mortality rate, 95%) [24, 27]. In fact, pregnancy is thought to be associated with more than half of all SPAA ruptures [46, 47]. Portal hypertension is an additional significant risk factor for rupture [24, 28, 29]. All symptomatic and ruptured aneurysms require repair, but intervention should also be considered for SAAs in patients who are pregnant or of childbearing age and may require liver transplantation [29]. This is the general consensus on the repair of pseudoaneurysms, regardless of size or symptoms in such patients. However, in the absence of these conditions, the indications for treatment are based mainly on the size of the aneurysm and the rate of growth. According to some recent studies, treatment is indicated for asymptomatic true SAAs larger than 25 mm in diameter [4, 12], whereas others suggest that intervention is indicated for SAAs larger than 20 mm in diameter [5, 48]. Although aneurysm neck ligation and aneurysmectomy with reconstruction are sometimes performed for SAAs in the main trunk of the splenic artery, arterial reconstruction is unnecessary, because the spleen has abundant collateral circulation via the short gastric arteries; thus, coil embolization by EVT, which has a high success rate and less risk of ischemia, may be appropriate (Fig. 2) [48]. Stent graft placement is challenging in a distal tortuous artery, but it may be appropriate for proximal SAAs (Fig. 5). For SAAs in the vicinity of the splenic hilum (laparoscopic), splenectomy is often performed.

#### Hepatic artery aneurysms

HAAs are the second most common VAA, accounting for 4–30% according to recent studies [5, 7, 12]. The distribution of 163 HAAs reported by Stanley et al. [10] in 1970 indicated that 80% were extrahepatic and 20% were intrahepatic, with approximately 60% of the extrahepatic artery aneurysms involving the common hepatic artery. HAAs are found more frequently in men. No relationship has been found between the morphology of the aneurysm and pregnancy. The frequency of rupture is reported as 20–30%, and the mortality rate after rupture is relatively high, at 35% [49]. In the recent literature, HAAs were the most frequently reported aneurysm of the visceral artery. Approximately, half of these HAAs were pseudoaneurysms located in the parenchyma of the liver, which reflects the increasing incidence of iatrogenic etiology for hepatic pseudoaneurysms, such as percutaneous biliary procedures, liver transplantation, and nonoperative management for blunt abdominal trauma. Atherosclerosis is the leading cause of HAA formation. Other less common causes include fibromuscular dysplasia, SAM, polyarteritis nodosa, infection, trauma, and biliary diseases [19, 25]. The incidence of pseudoaneurysms is rising with the number of percutaneous or endoscopic procedures and transarterial chemoembolizations. Most HAAs are asymptomatic and found incidentally during screening for other diseases. Prodromal symptoms of rupture rarely occur. Moreover, if rupture occurs within the bile duct, the patients may present with bloody vomit and stool from hemobilia, as well as anemia and jaundice. Treatment is generally indicated for all symptomatic HAAs, pseudoaneurysms, and aneurysms larger than 20 mm in diameter. Multiple HAAs and nonatherosclerotic aneurysms should also be treated, regardless of size, since these HAAs have a high risk of rupture [50]. Moreover, since rupture of extra-parenchymatous HAAs has a high mortality rate, treatment should be considered once these aneurysms are diagnosed [7, 22]. Like other VAAs, treatment options for HAAs depend on the anatomy, morphology, and location of the aneurysm, as well as the patient’s clinical status and comorbidities. Since the hepatic artery system includes a large number of anatomical variations, it is essential that the vessel running course and collateral routes are confirmed via angiography prior to treatment. If an aneurysm develops in the common hepatic artery, surgical ligation or endovascular coil embolization can be performed because the GDA facilitates collateral circulation to the liver. When the aneurysm is in the proper hepatic artery or the left or right hepatic artery, peripheral to the GDA branch, arterial reconstruction using either an autologous vein or a prosthetic conduit is generally required to maintain hepatic blood flow. In such cases, it is preferable to use an autologous vein graft because of the differences in diameter of the anastomosis site and infection resistance, but sufficient caution must be exercised to prevent compression, bending, or twisting of the graft by the organ or mesentery. The arteries that can be used for proximal anastomosis for bypassing the extrahepatic artery include the aorta, iliac artery, splenic artery, and renal artery. The surgical technique of reno-hepatic artery bypass, when bypassing from the right renal artery, is simple and effective, and the bypass distance is short [51]. Once the anatomical conditions, such as a relatively large arterial diameter and sufficient proximal and distal sealing zones, are confirmed, stent graft placement is indicated. End-to-end anastomosis with the omental artery is an option for reconstruction of the right or left extrahepatic artery. In practice, the right and left hepatic arteries have intrahepatic communication; thus, major complications arising from the use of coil embolization via EVT or ligation of one of the hepatic arteries (right or left) are rare. Most intrahepatic artery aneurysms are treated with embolization using EVT (Fig. 3), but hepatectomy is sometimes required.

#### Superior mesenteric artery aneurysms

**Fig. 7:**
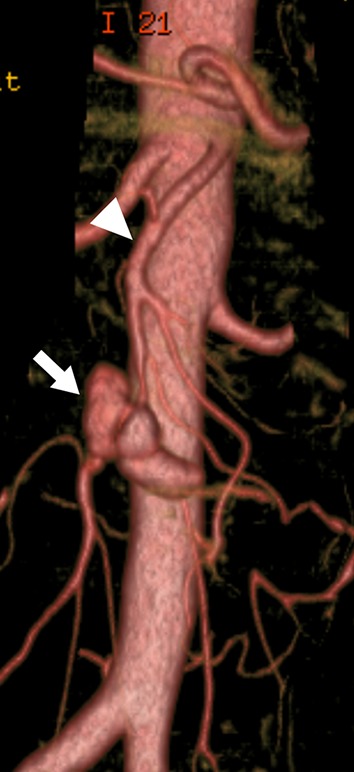
Three-dimensional volume rendered CT angiography shows an infected aneurysm (arrow) of the superior mesenteric artery (SMA) secondary to infectious endocarditis. Arrowhead shows the main trunk of the SMA

SMAAs account for 3–9% of all VAAs [5, 7, 12], and they are more common in men [52, 53]. Although infection was previously thought to be the main cause of SMAAs, in recent years, vascular wall degeneration, inflammation of the surrounding arteries such as that related to pancreatitis, or trauma, have been identified as more likely causes. A recent study of 21 SMAAs found that the cause was infection in only one case (5%), whereas it was atherosclerosis in two (10%), cystic medial dysplasia in two (10%), collagen vascular disorder in two (10%), polyarteritis nodosa in two (10%), and unknown in the remaining 11 (52%) [52]. Infected (mycotic) aneurysms are usually secondary to bacterial endocarditis and often affect individuals younger than 50 years old (Fig. 7). Although some SMAAs are asymptomatic, they are more often symptomatic than other types of VAA. Symptoms vary and include epigastralgia, abdominal angina, gastrointestinal bleeding, and vomiting. In some cases, SMAAs not only rupture but also lead to thrombosis and in turn, intestinal ischemia. The rates of SMAA rupture ranged from 38 to 50%, with an associated mortality of 30–90% [52, 54]. These high mortality rates may be due to free rupture into the peritoneal cavity with accompanying intestinal ischemia. When aneurysm expansion and associated dissection or progressive intraluminal thrombus progress beyond the branch of the inferior pancreaticoduodenal artery or the middle colic artery from the origin of the SMA, to reach the peripheral region, the peripheral SMA may become isolated from the collateral circulation routes from the celiac and inferior mesenteric arteries, resulting in intestinal ischemia from reduced blood flow to the SMA. Spontaneous isolated SMA dissection, which is rare, can also be associated with aneurysmal dilatation [55]. Although evidence is limited, recent studies suggest that since SMAAs have a higher risk of rupture and associated mortality, even when the patient has a good systemic condition, treatment is indicated regardless of the size of the aneurysm [52, 54]. All pseudoaneurysms and those with infectious (mycotic) etiology should also be repaired, because of the high risk of rupture and subsequent mortality. Since the 1953 report by DeBakey et al. of successful surgical treatment of SMAAs with ligation and division of the proximal and distal sites of the aneurysm [3], aneurysmorrhaphy and simple ligation have become the most common surgical procedures. Current treatment options for SMAAs include open surgery such as aneurysmectomy, aneurysmorrhaphy, simple ligation with or without arterial reconstruction, or EVT with coil embolization or stent graft exclusion. In open surgery, the need for further revascularization following simple ligation of the SMA depends on the collateral circulation from the pancreaticoduodenal and middle colic arteries through the GDA. Prior to concluding surgery, the viability of the intestinal tract should be carefully observed to decide if vascular reconstruction is required. Second-look surgery is sometimes performed. Endovascular embolization is particularly useful if aneurysms are distal to the origin of the SMA and have a small neck and good collateral flow [20]. Although long-term follow-up data are lacking, stent graft placement to exclude the aneurysm may be acceptable if there are adequate proximal and distal landing zones in high-risk patients with a hostile abdomen and/or severe comorbidities [56, 57].

#### Celiac artery aneurysms

CAAs account for only 4% of all VAAs; however, recent studies have reported that the number of patients with CAAs is increasing and includes those under follow-up observation [5, 7, 12]. CAAs tend to coexist with aortic aneurysms (18%) and other visceral aneurysms (38%) [58–60]. They are slightly more common in men, and the mean age of patients is 64 years old [58]. Historically, CAAs were often associated with syphilis or tuberculosis, whereas now, CAAs are associated with atherosclerosis, medial degeneration, trauma, and spontaneous celiac artery dissection [19]. CAA can also occur in patients with median arcuate ligament syndrome, as a result of extrinsic compression causing post-stenotic dilatation from altered flow mechanics, with retrograde flow through the pancreatic arcades [60]. Although rare, there have also been reports of CAA in patients whose celiac and superior mesenteric arteries have a common origin in the aorta (common celiomesenteric trunk) [61, 62]. As most cases progress without symptoms and are only diagnosed incidentally, the natural history of CAAs is not well understood. According to the literature, although rupture is relatively rare (10–20%), the mortality rate associated with CAA rupture is at least 50% [25]. With regard to the indications for elective surgery, since the surgical outcomes are relatively good, some studies recommend that immediate treatment be considered on discovery of the aneurysm; however, others advocate using the rule of thumb that surgery is indicated only when the aneurysm is at least twice the size of the diameter of the host artery. Notably, a recent study suggested that surgery is indicated after a comprehensive evaluation finds the aneurysm to be at least 25 mm in size [4]. The appropriate treatment approach is based only on the anatomy and location of the aneurysm, the urgency of intervention (ruptured or symptomatic), and the patient’s comorbidities. Occlusion of the celiac artery by coil embolization as treatment for CAA is effective, with less risk of organ ischemia, and may be indicated, especially for patients at high risk for open surgery [63, 64]. However, EVT is not applicable if the aneurysm involves the origin of the celiac artery, as there is no seal zone for a stent graft or a proximal space for coils. Surgical treatments include aneurysm resection and ligation of the celiac artery when it is possible to establish collateral blood flow from the GDA via the SMA or from the short gastric arteries. When arterial reconstruction is required, bypass surgery from the aorta using a prosthetic vascular graft or autologous vein graft is performed.

#### Pancreaticoduodenal and gastroduodenal artery aneurysms

**Fig. 8:**
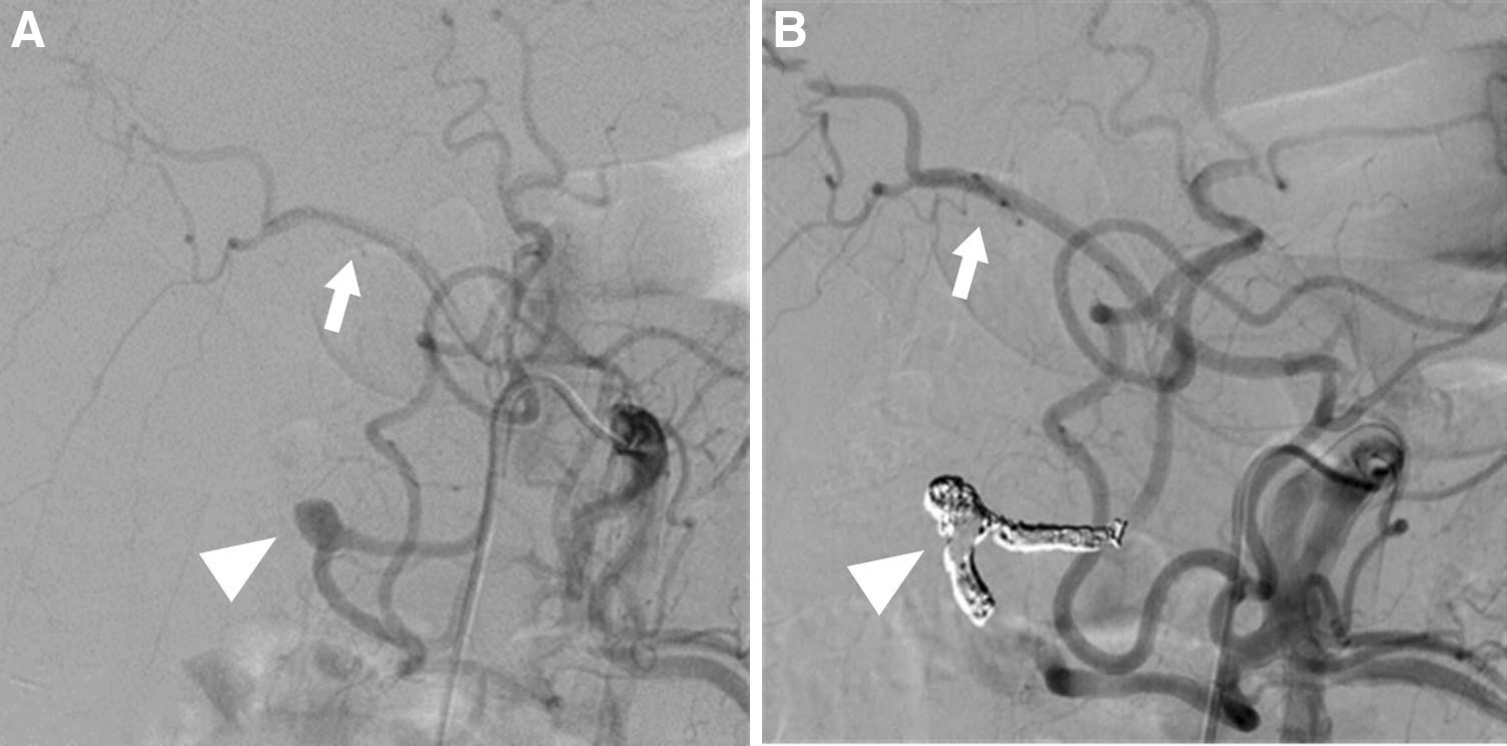
Pancreaticoduodenal artery aneurysm in 54-year-man. **a** Selective angiography of the superior mesenteric artery shows a pancreaticoduodenal artery aneurysm (arrowhead) in a patient with median arcuate compression of the celiac artery. The hepatic artery (arrow) were visualized through the collateral flow via the pancreaticoduodenal arcade. **b** Selective angiography of the superior mesenteric artery shows successful coil embolization with microcoils (arrowhead) and continued filling of the hepatic artery (arrow) through the collaterals

**Fig. 9:**
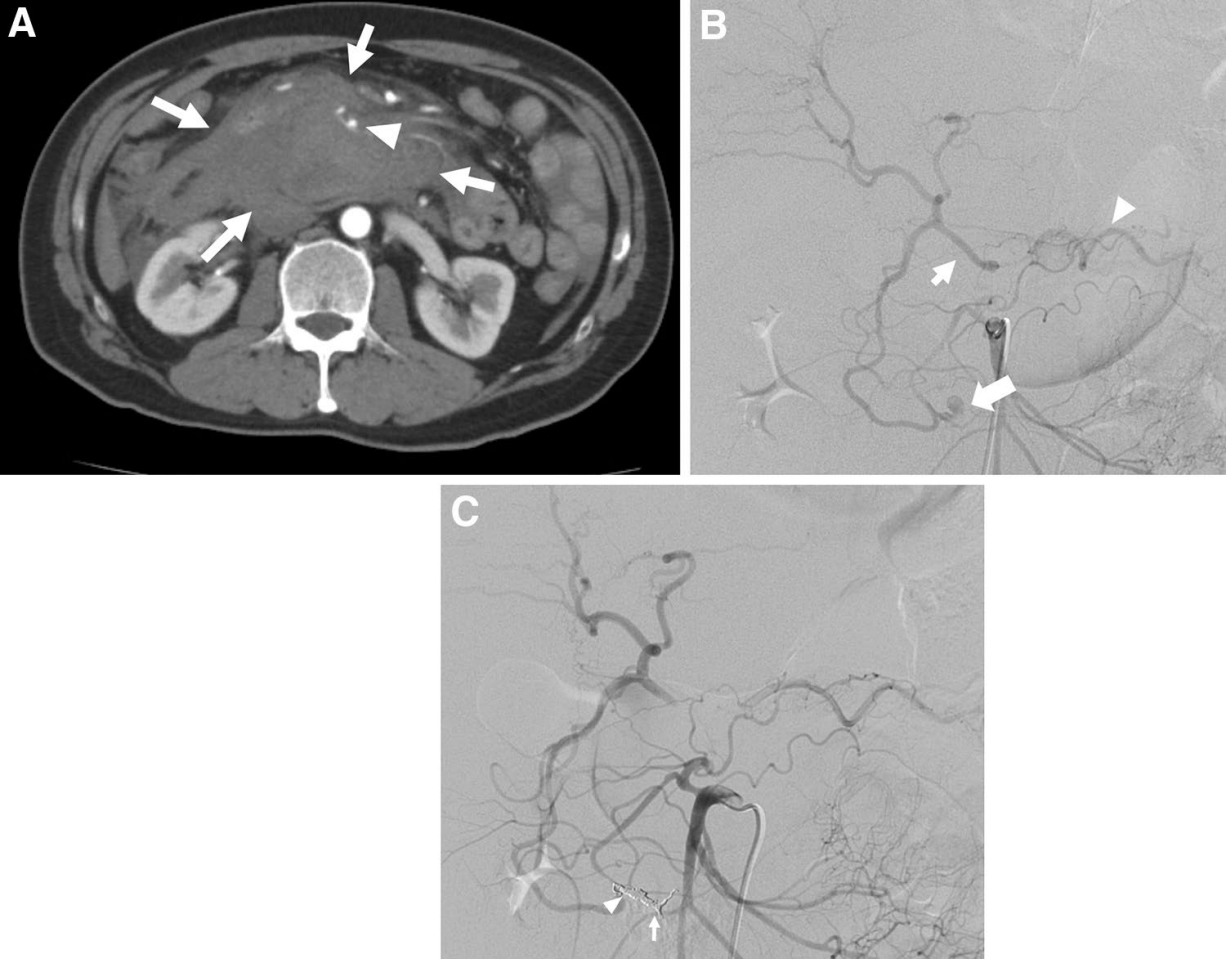
Pseudoaneurysm in a branch of the pancreaticoduodenal arcade. **a** Arterial phase contrast-enhanced CT scan shows a massive retroperitoneal hematoma (arrow) surrounding the pancreatic head. A pseudoaneurysm (arrowhead) is visualized in the hematoma. **b** Selective superior mesenteric digital subtraction angiography shows visualization of the hepatic (arrow) and splenic arteries (arrowhead) via collateral circulation, suggesting severe stenosis or occlusion of the celiac artery. The ruptured aneurysm (large arrow) can be seen in the branch of pancreaticoduodenal arcade. **c** Embolization using metallic coils of the distal site of the aneurysm (arrow) and the proximal site of the aneurysm (arrowhead) resulted in complete embolization of the pseudoaneurysm with an isolation technique

Historically, PDAAs, GDAAs, and aneurysms of their branches are extremely rare, accounting for only 2% and 1.5% of all VAAs, respectively [16]. Pseudoaneurysms are more frequent than true aneurysms and are usually associated with pancreatitis or trauma, including complications of pancreatic surgery [65]. In clinical practice, the frequency of pseudoaneurysms around the pancreatic arcade is considered to be higher. Other proposed causes include true aneurysm formation resulting from increased blood flow velocity after celiac artery and/or SMA occlusion or stenosis (Fig. 8) [66–69]. Recent studies report cases of pseudoaneurysm rupture caused by SAM [70, 71]. Asymptomatic aneurysms are rarely diagnosed and usually found only when the patient complains of epigastralgia or pain radiating to the back in accordance with rupture (Fig. 9). In fact, over half of the cases reported involve rupture, and the frequency of gastrointestinal or biliary tract bleeding and bleeding into the retroperitoneal or intraperitoneal space are about the same [72]. Among 90 patients with PDAAs, 62% presented with rupture, resulting in a 21% mortality rate [72]. In this series, there was no correlation between aneurysm size and risk of rupture, and all aneurysms smaller than 1 cm had already ruptured at presentation [72]. In another series, nearly 50% of patients with true PDAAs presented with a ruptured aneurysm, regardless of size, resulting in a 26% mortality rate [66]. The rupture rate for GDAAs was also high (35%), with an associated mortality rate of 40% [65, 72]. Since both rupture rates and mortality rates following rupture are high for PDAAs and GDAAs regardless of the aneurysm size, an accurate image-based diagnosis is essential and immediate therapeutic intervention is required. The lesion surrounding an aneurysm can be greatly affected by pancreatitis or following pancreatic surgery. In most cases, a surgical approach for these lesions is difficult. Therefore, endovascular coil embolization or stent graft placement is usually performed as these procedures have fewer associated complications and better outcomes than open surgery (Figs. 8, 9) [13, 33, 65, 72]. Open surgery is performed in an emergency if EVT is not indicated, or has failed, or the medical facility has no EVT specialists. When an association between increased collateral flow and aneurysm formation is identified in patients with CA occlusive disease, CA revascularization, including EVT, bypass, or release of the median arcuate ligament, in addition to the aneurysm treatment, may be an option to prevent recurrence or further aneurysm formation [68, 69].

#### Gastric, jejunal, and gastroepiploic aneurysms

GAAs and GEAAs account for less than 4% of all VAAs [73]. The frequency of GAA onset is approximately 10 times higher than that of GEAAs [74], but both are managed in the same manner. They are common among men and are rarely detected early due to their asymptomatic nature. Hence, a diagnosis is made after patients complain of abdominal pain, gastrointestinal bleeding, or rupture [73]. The mortality rate following aneurysm rupture is as high as 70% [74]. The objective of treatment for rupture is bleeding control, and depending upon the specific circumstances, either open surgery or EVT is performed. According to the literature, open surgery is the primary procedure utilized, but studies reporting the use of selective coil embolization have been increasing [75, 76].

#### Inferior mesenteric, jejunal, ileal, and colic artery aneurysms

IMAAs are extremely rare, accounting for less than 1% of all VAAs. In fact, only 40–50 cases of IMAA have been reported [77]. Approximately, half of all IMAAs involve atherosclerosis and are related to SMA and CA occlusion [77]. In a recent literature review, 11 of 54 patients with IMAAs had a ruptured aneurysm and 4 of these patients died [77].

Jejunal, ileal, and colic artery aneurysms rarely occur and account for 3–6% of all VAAs [5, 7, 12]. Most reported lesions are solitary and smaller than other VAAs, being about 10 mm in diameter [78, 79]. Among mesenteric branch artery aneurysms, colic artery rupture is commonly reported, and the mortality rate associated with ruptures is 20% [16]. Most patients who suffer rupture have an aneurysm size of less than 1 cm. Thus, even if asymptomatic, treatment is recommended regardless of aneurysm size [78]. Although the most common therapeutic strategy is open surgery, there is a report stating that EVT was effective for rupture [79].

## Conclusion

We reviewed the current literature on VAAs and their management based on location of origin, cause, and treatment. The recent advances in EVT have resulted in this modality being used increasingly as an alternative to open surgery. With the increasing popularity of hybrid operating rooms, a more flexible approach, including a hybrid approach, is being employed. EVT is now being performed for rupture initially, with conversion to open surgery, if necessary. Performing open surgery as the initial option is decreasing; however, to preserve organ blood flow, surgical vascular reconstruction remains an important treatment option. Every patient with a VAA requires an appropriate treatment strategy that prioritizes organ blood flow preservation, based on an accurate imaging diagnosis of the vascular anatomy, with a good understanding of their general condition, comorbidities, and risks.
