# Correction to: Current management strategies for visceral artery aneurysms: an overview

**DOI:** 10.1007/s00595-019-01947-x

**Published:** 2020-01-08

**Authors:** Hideaki Obara, Matsubara Kentaro, Masanori Inoue, Yuko Kitagawa

**Affiliations:** 1 Department of Surgery, Keio University School of Medicine, 35 Shinanomachi, Shinjuku-ku, Tokyo, 160-8582 Japan; 2 Department of Diagnostic Radiology, Keio University School of Medicine, Tokyo, Japan

## Correction to: Surgery Today (2020) 50:38–49 10.1007/s00595-019-01898-3

The article Current management strategies for visceral artery aneurysms: an overview, written by Hideaki Obara, Matsubara Kentaro, Masanori Inoue and Yuko Kitagawa, was originally published Online First without Open Access. After publication in volume 50, issue 1, pages 38–49 the author decided to opt for Open Choice and to make the article an Open Access publication. Therefore, the copyright of the article has been changed to © The Author(s) 2020 and the article is forthwith distributed under the terms of the Creative Commons Attribution 4.0 International License (https://creativecommons.org/licenses/by/4.0/), which permits use, duplication, adaptation, distribution and reproduction in any medium or format, as long as you give appropriate credit to the original author(s) and the source, provide a link to the Creative Commons license, and indicate if changes were made.

The original article has been corrected.

